# Smoking cessation among people with mental illness: A South African perspective

**DOI:** 10.4102/safp.v64i1.5489

**Published:** 2022-08-30

**Authors:** Tejil Morar, Lesley Robertson

**Affiliations:** 1 Department of Psychiatry, Faculty of Health Sciences, School of Clinical Medicine, University of the Witwatersrand, Johannesburg, South Africa

**Keywords:** smoking, tobacco, smoking cessation, mental illness, South Africa

## Abstract

Tobacco use is recognised as a serious, worldwide public health concern. Smoking cessation is of great interest across a wide range of medical specialities, including family medicine. However, smoking cessation among people with mental illness (PWMI) has attracted scant attention in South African literature. This is despite PWMI suffering disproportionately from the damages of tobacco. The harms of smoking are not limited to physical health but extend to mental health. This article discusses the need for multifaceted smoking cessation treatments for PWMI in the public health sector, taking into consideration the prevalence and unique drivers of smoking in this population. A brief overview of patterns of tobacco use, associated harms and smoking cessation interventions in South Africa is given; all within the context of mental illness.

## Introduction

Smoking cessation is of great interest across a wide range of medical specialities, and the World Health Organization (WHO) aims to reduce the global prevalence of tobacco use by 30% between 2010 and 2025.^1^ Although tobacco use occurs worldwide, the highest prevalence is in low- and middle-income countries (LMICs). Of concern, South Africa has made little progress in reducing tobacco use over the past decade and is unlikely to achieve a 30% reduction in prevalence.^1^ In South Africa, tobacco use prevalence among people aged 15 years and older was estimated at 21.0% (34.1% and 8.4% among men and women, respectively) in 2010, 20.2% in 2020 and is projected to be 19.7% in 2025.

While smoking rates have decreased in the past decade among the general population globally, most likely as a result of tobacco control policies, rates among people with mental illness (PWMI) remain relatively stable.^2^ Even where tobacco control policies are advanced and successful, PWMI are often left behind and are noted to consume 44% of all cigarettes in developed countries.^3^ Exclusion of this population from smoking cessation strategies is likely to have major consequences.

In South Africa, the 12-month prevalence of common mental disorders was found to be 16.5% in 2004.^4^ People with common and severe mental disorders access the health system via primary care for both physical and mental health complaints. Given the high rates of comorbidity between smoking and mental illness (see Table 1), screening for mental disorders among people who smoke in the primary health care setting may allow for earlier intervention, adaptation of smoking cessation interventions and improved chances of smoking cessation.^5^ Similarly, addressing tobacco use among people seeking treatment for mental disorders should improve physical health outcomes.

**TABLE 1:** Prevalence of mental disorders and tobacco use among people with mental illness.

Mental disorder	Estimated prevalence of mental disorder	Estimated comorbidity with tobacco use	Comments
Attention-deficit/hyperactivity disorder (ADHD)	Bakare^13^: 5.4% – 8.7% of school children in Africa	McClernon et al.^14^: ADHD has an estimated 40.0% comorbidity with tobacco smoking	People with ADHD begin smoking at an earlier age (often adolescence) and are at a higher risk of subsequently developing a tobacco use disorder.^15^ These individuals appear to be dependent on smoking by their early twenties. A meta-analysis conducted by Schoenfelder et al. concluded that stimulant treatment of ADHD may lower the risk of smoking.^16^
Post-traumatic stress disorder (PTSD)	Herman et al.^4^: 2.3% lifetime prevalence in South Africa	Pericot-Valverde et al.^17^: The comorbidity of smoking in patients with PTSD is approximately 45.0%	PTSD and smoking both contribute to higher rates of mortality, independently as well as cumulatively.^17^ Addressing tobacco use disorders may improve morbidity and mortality in this population.
Anxiety disorders	Herman et al.^4^: 15.8% lifetime prevalence in South Africa	Garey et al.^18^: The smoking rate of adults diagnosed with any anxiety disorder is 33.4%	Anxiety may act as a barrier to smoking cessation, with smokers with anxiety having anticipatory fear of coping without cigarettes, relapsing following cessation and worrying about mood changes during cessation attempts.^18^ These fears appear to translate into a worsened subjective experience of tobacco withdrawal and a greater risk of relapse. Anxiety is recognised as one of the most common signs of tobacco withdrawal in general, possibly an added burden in patients with anxiety disorders.
Major depressive disorder (MDD)	Herman et al.^4^: 9.8% lifetime prevalence in South Africa	Prochaska et al.^6^: Approximately 40% of persons diagnosed with MDD utilise tobacco, and 60% of smokers enrolled in smoking cessation programmes have a diagnosis of MDD.	Depression is a risk factor for tobacco use and failed quit attempts.^6^ The converse is also applicable.^6^ Clinical applications of these findings include the need to screen for depression in persons who do not respond to standard smoking cessation strategies and primary preventative measures regarding tobacco use in individuals newly diagnosed with depression.
Bipolar disorder	Esan et al.^19^: 0.1% – 1.83% lifetime prevalence in Africa	Evins et al.^20^: Up to 75% of patients with bipolar disorder smoke cigarettes regularly, yet few smoking cessation trials have been conducted among this population.	Failure rates of these individuals in standard smoking cessation programmes appear higher, indicating unmet needs of these patients.^20^
Schizophrenia	Simeone et al.^21^: 0.75% lifetime prevalence in Africa	Cather et al.^22^: 79% of patients with schizophrenia spectrum disorders smoke regularly.	People with schizophrenia are a vulnerable group, exposed to various risk factors for initiation of smoking.^22,23^ These include poverty, a lower level of education, unemployment, peer influences, cigarette availability, stress and impulsivity. Smoking is maintained by the effects of nicotine on negative symptoms and the potent reinforcing effects of nicotine. Alternative reinforcers on the reward pathway appear less effective in these patients. Quitting attempts may not be initiated due to insufficient treatments or clinical involvement, limited coping strategies and apprehension around withdrawal. Unsuccessful attempts can be attributed to the effects of nicotine withdrawal, intolerance of discomfort or anxiety sensitivity, poor social support and the conception of relapse as failure.
Other substance use disorders	Herman et al.^4^: 13.3% lifetime prevalence in South Africa	Degenhardt et al.^24^: Compared to people who have never smoked, tobacco users are: Five times more likely to have an alcohol use disorder. Five and a half times more likely to have a sedative, stimulant, or opiate-use disorder. Nine times more likely to have a cannabis use disorder.^24^	Previous theories that targeting smoking cessation in people with untreated substance use disorders is harmful have been refuted.^24^ Treating both tobacco and other substance use disorders concurrently will most likely result in an improvement in both conditions.
Personality disorders (PD)	Suliman et al.^25^: 6.8% in South Africa (multiple imputation prevalence estimate)	Zvolensky et al.^26^: Current smoking is associated with significantly lower odds of having avoidant or obsessive-compulsive PD. However, current smoking was associated with increased odds of antisocial PD. No statistically significant relationship between current smoking and dependent, paranoid, schizoid or histrionic PD was found. The study did not include patients with borderline PD.	Antisocial PD appears to have the highest association with tobacco use compared to avoidant, obsessive-compulsive, dependent, paranoid, schizoid or histrionic PD. Approximately 63% of patients with antisocial PD compared to 17% of those without the disorder are currently nicotine dependent (point prevalence).^26^

*Source:* Please see the full reference list of the article Herman AA, Stein DJ, Seedat S, et al. The South Africa stress and health (SASH) study: 12-month and lifetime prevalence of common mental disorders. S Afr Med J. 2009;99:339–344, for more information.

## Epidemiology of smoking among people with mental illness

**FIGURE 1:**
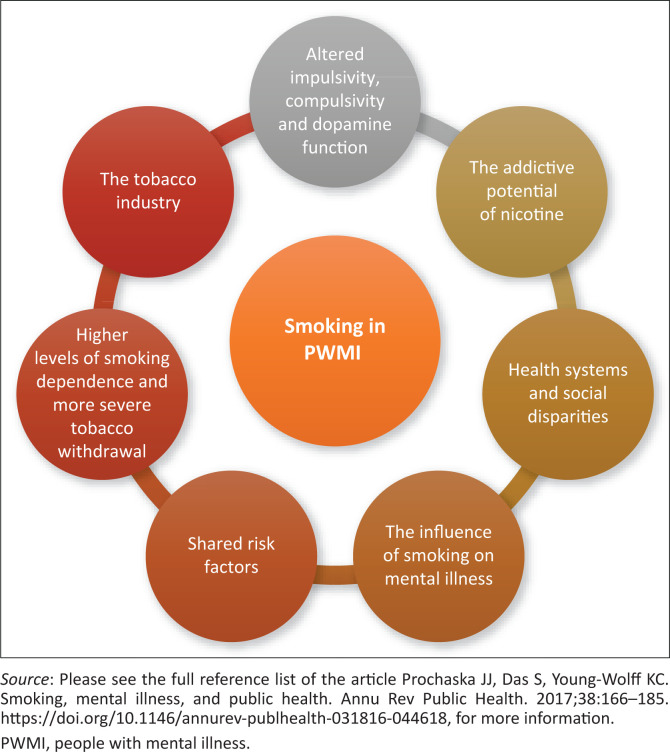
Factors influencing the relationship between mental illness and smoking.

The multifactorial and bidirectional relationship between smoking and mental illness is illustrated in Figure 1. Smoking increases vulnerability to mental illness, and mental illness increases the likelihood of smoking.^3^ Globally, smoking rates among PWMI are two to three times higher than the general population, with greater severity observed in terms of amount and chronicity of tobacco use.^3,6^ A 2007 population-based study in Australia and the United States (US) found the 12-month prevalence of mental disorders among smokers to be 31.7% (US) and 32.4% (Australia), considerably higher than 20.0% found among the general population.^7^

South African studies reiterate higher smoking rates in PWMI compared to the general population. In one of the few studies conducted in South Africa among PWMI by Tindimwebwa et al. in 2020 at the Cecilia Makiwane Hospital in Eastern Cape, 59.4% of 390 outpatient participants reported using tobacco at some point in their lives.^8^ The lifetime prevalence of tobacco use differed in various disorders: cannabis-induced disorders (97.3%), schizophrenia (67.9%), bipolar and related disorders (43.5%) and major depressive disorders (36.1%).^8^ These rates are even higher among psychiatric inpatients. In a study conducted among 116 inpatients at Stikland Hospital in the Western Cape, Du Plooy et al.^9^ found that 91.4% were current smokers. Almost half (46.6%) of the participants were diagnosed with schizophrenia, 22.4% with substance-induced psychotic disorder and 12.1% with bipolar disorder.^9^

Coronavirus disease 2019 (COVID-19) has had varying effects on tobacco use worldwide, with increased quit rates in some countries as people are concerned about their health, and disruption of tobacco cessation campaigns in others.^1^ During the COVID-19 pandemic, the South African government imposed a ban on the sale of tobacco products between 27 March 2020 and 17 August 2020 without any additional smoking cessation support. Notably, a large online survey^10^ found that 93% of smokers continued to purchase cigarettes through informal channels despite the sales ban.

Tobacco use is noted to be a risk factor for contracting COVID-19 and a more severe course of illness.^11^ In addition, mental disorders are associated with higher COVID-19-related mortality, after adjusting for smoking and other clinical risk factors.^12^ Individuals with schizophrenia and bipolar disorders appear most vulnerable. Thus, PWMI who smoke may be a particularly high-risk group in the pandemic.

## The relationship between smoking and mental illness

Various factors influence the high rates of comorbidity between mental illness and tobacco use, as depicted in Figure 1.

### Shared risk factors

Possible shared risk factors include genetics, personality traits, neurobiology and environmental stressors.^17^ Smoking initiation and dependence have been associated with the COMT Val^158^Met polymorphism in at least four gene-association studies.^27^ This COMT Val^158^Met polymorphism, which affects catecholamine metabolism, is also involved in the pathophysiology of several mental disorders, including schizophrenia.^28^ Epigenetic mechanisms during critical developmental periods may also increase the risk for both mental illness and addiction.^29^ In adolescence, attentional and emotional disturbances are linked to internalising and externalising behaviour problems and to smoking.^30^ Regarding underlying neurobiological mechanisms, the mesolimbic dopamine pathway has been implicated in both the positive symptoms of schizophrenia as well as addiction.^31^ In terms of environmental stressors, traumatic events are associated with smoking as well as the aetiology of mental illnesses, especially post-traumatic stress disorder.^32^

### The addictive potential of nicotine

Properties which increase the addiction potential of a substance include a shorter half-life and a rapid onset of action.^31^ Among the general population, the probability of nicotine dependence following use of tobacco at least once is 32%. This is higher than the probability of dependence following heroin (23%), cocaine (17%), alcohol (15%), other stimulants (11%), anxiolytics (9%) or cannabis (9%) use.^31^

Among people without mental illness, nicotine dependence is associated with upregulation of nicotinic receptors.^33^ This is proportionate to the number of cigarettes smoked and reverses after smoking cessation. However, among people with schizophrenia, nicotinic receptors in the hippocampus, cortex, thalamus and caudate nucleus do not show upregulation with chronic tobacco use or downregulation upon cessation. Additionally, dysfunctional nicotinic receptors have been implicated in the pathogenesis of schizophrenia.^29,33^

### The influence of smoking on mental illness

The psychoactive effects of smoking are predominantly driven by nicotine, which may initially alleviate symptoms of depression and anxiety symptoms.^30^ However, once nicotine dependence occurs, the risk of negative affectivity is increased. Nicotine is also associated with other substance use disorders. Population-based studies suggest that nicotine may predispose users to cannabis and cocaine use, possibly related to priming of the nucleus accumbens and enhancement of the effects of other drugs.^34^ Among South African adolescents in Johannesburg, Pahl et al.^35^ found that the severity of nicotine dependence predicted violent or deviant behaviour, illicit drug use, binge-drinking of alcohol, early sexual intercourse, multiple sexual partners and unsafe sex practices.

A further difficulty among PWMI is that smoking may reduce the efficacy of psychotropic medication, such as antidepressants and antipsychotics, possibly worsening treatment outcomes.^31^

### Altered impulsivity, compulsivity and dopamine function

Addiction should not be attributed to lack of willpower or poor upbringing, as it has distinct underlying neurobiological pathological processes.^31^ Nicotine results in an increase in dopamine in the ventral striatum (also known as the nucleus accumbens or reward pathway) and serves as potent positive reinforcement that drives a compulsive habit. These processes may be conceptualised by the ‘top-down and bottom-up’ model.^31^ Bottom-up stimulation occurs as impulsivity and compulsivity. Top-down inhibitory control of impulsivity arises from the prefrontal cortex.^31^

People with mental illness may have excessive bottom-up stimulation with relatively less top-down control.

Impulsivity refers to acting with a lack of foresight and an inability to inhibit risky behaviour.^31^ People with mental illness may be more impulsive due to hyperactivity in a circuit projecting from the ventral striatum to the thalamus, from the thalamus to the ventromedial prefrontal cortex (VMPFC) and from the VMPFC back to the ventral striatum.^31^

Compulsivity can be defined as an inability to halt ongoing actions.^31^ Compulsions are conditioned responses to stimuli, outside the bounds of voluntary control and intensified by positive reinforcement (e.g. pleasure) or negative reinforcement (e.g. relief of withdrawal or craving). These are driven by a circuit involving the dorsal striatum, thalamus and orbitofrontal cortex, which may be overactive in PWMI.^31^

### Health systems and social disparities

There are minimal dedicated, clinic- and hospital-based pharmacological smoking cessation centres in South Africa and virtually none customised for PWMI.^36^ Furthermore, there is a lack of randomised controlled trials regarding smoking cessation interventions among PWMI in South Africa. This makes it difficult to implement evidence-based strategies to quit smoking in this population. Generally, the confounding factor of mental illness is not considered sufficiently in South African research relating to smoking. In 2015, Reddy et al. published results relating to tobacco use from the first South African National Health and Nutrition Examination Survey (SANHANES).^37^ Factors affecting quit attempts in current smokers were explored; however, data on the influence of psychological factors was noticeably absent. This is despite the SANHANES including the Kessler-10 scale measuring psychological distress.^38^

Stigma, including beliefs that PWMI cannot or will not quit smoking as it is ‘all they have’, may also contribute to the neglect of PWMI in smoking cessation interventions; despite robust evidence of disproportionate harm to these individuals from tobacco.^39^ It has been postulated that smoking offers PWMI a sense of identity and encourages social interaction in an otherwise marginalised group.^9^ There are also concerns that inpatient psychiatric environments may promote smoking behaviour by peer pressure, boredom, provision of cigarettes as incentives or allaying patient agitation with cigarettes.^6,9^

Psychiatrists have been criticised for the lack of implementation of smoking cessation strategies in PWMI. In an American survey of over 3000 physicians, psychiatrists were the least likely to address tobacco use with patients, with only 23.0% providing assistance with quit attempts and 11.0% providing treatment referrals.^6^

Both mental illness and smoking are associated with deprivation, further perpetuating the mental health–poverty cycle.^39,40^

### Higher levels of smoking dependence and more severe tobacco withdrawal

Patients with schizophrenia smoke more cigarettes, take more frequent puffs over a shorter duration, extract more nicotine from a cigarette, suffer greater withdrawal and are more dependent on nicotine than those without mental illness.^6,9,22,41^ Ayers et al.^42^ found that patients with anxiety disorders were more likely to suffer neuropsychiatric adverse events during smoking cessation than those without. Du Plooy et al.^9^ found that 87.8% of male smokers in a South African psychiatric hospital smoked daily. The rate of active smokers in the hospital was three times that of the general population. The rate of high or very high nicotine dependency in these patients was 56% compared to less than 20% reported in the general population.^9^

### The tobacco industry

International studies confirm that the tobacco industry targets at-risk or susceptible populations, for example, the youth, socially deprived groups and certain racial or ethnic groups.^39^ International tobacco marketing is often based on mental health needs such as stress relief, cognitive arousal, productivity enhancement and weight control.^39^ Research into the tobacco industry’s approach to PWMI in South Africa is lacking.

### Self-medication hypothesis (SMH)

There is contradictory data surrounding evidence for the SMH of tobacco use. The SMH postulates that smoking relieves psychiatric symptoms, as well as the side effects of antipsychotic medications. While smoking may lower the intensity of negative symptoms of schizophrenia, it also appears to be associated with higher rates of physical aggression and positive symptoms.^30,43^ Smoking alleviates the side effects of antipsychotics because smoking lowers the plasma levels of antipsychotics via the cytochrome P450 system, thereby reducing both therapeutic and side effects. The SMH requires critical review and a more extensive evidence base to be confirmed.^43^

## The harms of tobacco use

Scientific literature consistently reiterates the plethora of health-related harms of tobacco use. 11.5 million people died from tobacco-related deaths in 2015.^3^ People with mental illness suffer disproportionately from the medical complications of smoking.^44^ Those suffering from severe mental disorders have a lower life expectancy than the general population, by a staggering 10–25 years.^45^ The majority of these deaths are attributable to chronic noncommunicable diseases such as cardiovascular and respiratory illnesses, for which smoking is a significant risk factor.^45^ In the 1950s, it was thought that the incidence of lung cancer among smokers with schizophrenia was lower than the general population. This has since been refuted and attributed more to these patients not living long enough to get cancer and a lower detection of cancer in these patients.^6^

In a large-scale population-based study by Callaghan et al. published in 2013, patients diagnosed with schizophrenia; bipolar disorder and depression all displayed an elevated risk of mortality from tobacco-related complications.^44^ Approximately half of deaths in patients hospitalised for schizophrenia and mood disorders were causally linked to tobacco.^44^ Tobacco also contributes significantly to mortality in patients with opioid use disorders.^6^ Tobacco compounds the physical complications of other substances, with a 38-fold increased risk of mouth and throat cancer in patients with both alcohol and tobacco use disorder.^6^ Combating tobacco use in PWMI should improve premature mortality in this population.

In addition to the physical complications of smoking in PWMI, there are financial consequences which can negatively impact treatment and life expectancy. In smokers with schizophrenia, the median spending on cigarettes is 27% of their income.^6^

## Nicotine disorders

Nicotine is widely recognised as the major addictive agent in all tobacco products.^46^ The WHO’s 11th International Statistical Classification of Diseases and Related Health Problems (ICD) is set to be implemented in January 2022.^47^ Various disorders due to nicotine use are listed in chapter 6C4A:

- episode of harmful use of nicotine
- harmful nicotine use
- nicotine dependence
- nicotine intoxication
- nicotine withdrawal
- other specified disorders due to nicotine
- unspecified disorders due to nicotine.

Fifty percent of individuals who quit smoking for two or more days meet the criteria for nicotine withdrawal.^41^ Nicotine withdrawal typically peaks at two to three days after abstinence and lasts for two to three weeks. There are debates around whether nicotine withdrawal can lead to the onset of a new mental disorder or relapse of an existing one, but if this occurs, it is certainly in the minority.^41^ Nicotine withdrawal can, however, mimic, intensify or disguise other disorders or adverse effects of medications. Reduction in symptoms with the use of nicotine medications confirms the diagnosis.^41^ One may argue that allowing individuals to experience withdrawal symptoms for up to three weeks without pharmacological support is a deviation from ethical practice.

Isolating nicotine from tobacco relieves tobacco withdrawal symptoms while offering less exposure to toxins and a lower risk of addiction.^46^ Tobacco withdrawal symptoms negatively impact the success of quitting attempts.^46^ Hence, it makes sense to target nicotine withdrawal symptoms with nicotine replacement therapy (NRT) in smoking cessation.

Generally, the path to smoking cessation is characterised by periods of relapse and remission.^48^ Many authors emphasise that an abstinence model of substance abuse treatment is difficult to achieve.^48^ Instead, efforts should focus on reducing consumption and harm reduction.^48^ Relapse should be viewed as an opportunity to gain more information around triggers, and so on, and increase the likelihood of future successful attempts rather than failure.

## Tobacco control policies and smoking cessation in people with mental illness

International commentaries argue that PWMI who smoke should be a priority group when it comes to scientific funding and treatment resources.^40^ Despite the robust associations between smoking and mental illness, PWMI have a motivation to quit smoking that is comparable to the general population.^49^ If motivation is lacking, motivational interviewing can initiate the cycle of change. Heavy and severe smoking among PWMI should not automatically result in exclusion from interventions.

Smoking cessation is associated with an improvement in mental health. A systematic review including over 26 studies concluded that smoking cessation is associated with a reduction in depression, anxiety and stress.^50^ In addition, it has been linked to an improvement in mood and quality of life compared to ongoing tobacco use. These effects appear as significant in PWMI as in those without, and they are surprisingly equally or more significant than those on antidepressant treatment for mood and anxiety disorders.^50^ This is a compelling argument to include smoking cessation in the management of mood and anxiety.

The WHO Framework Convention for Tobacco Control provides the MPOWER package, which are six cost-effective measures to implement tobacco control.^51^ These are listed in Table 2, accompanied by possible challenges of implementation among PWMI.

**TABLE 2:** MPOWER package and considerations among people with mental illness (PWMI).

MPOWER package	Considerations regarding PWMI
Monitor tobacco use and prevention policies	There is a paucity of data about smoking cessation in PWMI in South Africa, making it a challenge to monitor tobacco use and prevention policies.
Protect people from tobacco smoke	The minority of patients who do not smoke in psychiatric hospitals are often exposed to second-hand smoke as a result of hurdles in implementation of smoking bans within these hospitals.^52^
Offer help to quit tobacco use	Agaku et al. found that a minimal 26.1% of South African smokers had ever received tobacco cessation counselling from a trained professional, and only 3.9% had ever utilised nicotine replacement therapy.^53^ It is postulated that these numbers are even lower in PWMI.
Warn about the dangers of tobacco	Warning PWMI about the dangers of tobacco use may not be a deterrent, as impulsivity and compulsivity may override the prefrontal cortex.^31^
Enforce bans on tobacco advertising, promotion and sponsorship	These interventions may have less effectiveness in PWMI,^2^ but data in South Africa is sparse.
Raise taxes on tobacco	If PWMI continue to smoke following tax raises on tobacco, this may further exacerbate the known association between poverty and mental illness.^39,40^

*Source:* World Health Organization. MPOWER: A policy package to reverse the tobacco epidemic. MPOWER Un Plan Medidas Para Hacer Retroceder Epidemia Tab. 2008; p. 39.

PWMI, people with mental illness.

In South Africa, opportunities for smoking screening and cessation advice are largely overlooked in the primary healthcare setting. Omole et al. found that, during a 2008 study in a large South African Community Health Centre, 87.1% of participants were not screened for tobacco use during their current visit.^54^ In a 2010 qualitative study among South African clinicians by Omole et al., possible barriers identified included health system barriers (time constraints, inaccessibility of smoking cessation medications and ineffectual referral systems) and clinician limitations (perceived ineffectiveness of smoking cessation treatments and limited knowledge and skill on evidence-based interventions).^55^

Primary healthcare providers can reach 80% of tobacco users per year,^56^ providing an ideal opportunity to screen for mental illness. They can also precipitate a quit attempt in 40% of tobacco users and achieve successful cessation in 2% – 3%.^56^ Over 80% of smokers attempt to quit during their course of use, but 60% relapse within a week and less than 5% remain abstinent. However, most individuals make multiple attempts, resulting in 50% of users abstaining.^41^ In a 2016 study at a South African district hospital, Bokoro et al. found that stress and cravings were the main barriers to quit attempts,^57^ both of which can be addressed in primary healthcare. The clinical practice guideline on tobacco smoking cessation for South Africa published in the *South African Medical Journal* in 2013 acknowledges that successful smoking cessation interventions call for holistic management of patients, including exploring why an individual smokes (stress, depression etc.) as well as addressing the biological consequences of nicotine withdrawal.^58^ The approach which yields the highest chance of success is one that encompasses biological (e.g. reduction of cravings and withdrawal symptoms pharmacologically), psychological (e.g. motivational interviewing) and social support (e.g. support groups).^58^

The integrated model of care is considered the gold standard in the dual diagnosis of a mental disorder and substance use disorder.^59^ This model involves the same clinician or team of clinicians providing both mental health and addiction treatment in a coordinated manner. Specific treatment plans based on the integrated model of care include integrated dual disorder treatment (active and empathetic outreach, continuity of care, optimism, specific treatment to stage of change and involvement of family members) and assertive community treatment (services offered in the community, assertive engagement, high intensity services, smaller caseloads and continuity of a multidisciplinary team).^59^

## Pharmacologically assisted smoking cessation

For the general population, the British National Institute for Care and Health Excellence (NICE) recommends that every smoker should be offered support in smoking cessation^60^ and recommends three first-line smoking cessation medications: (1) NRT, (2) varenicline and (3) bupropion.

These pharmacological smoking cessation treatments may also reduce smoking relapse rates among PWMI without incurring adverse psychiatric effects.^23^ However, guidelines for smoking cessation among PWMI are limited. The 14th edition of the *Maudsley Prescribing Guidelines*, which is informed by the NICE guidelines, recommend that PWMI who feel unable or unwilling to quit should be provided with treatment to abstain while in a hospital setting.^46^ They suggest that NRT and e-cigarettes may be encouraged as a form of harm reduction even if smoking continues, as tobacco consumption may be reduced. Among PWMI who wish to quit smoking, the *Maudsley Prescribing Guidelines* recommend using NRT or varenicline as first-line treatment and bupropion as second-line. The *Maudsley Prescribing Guidelines* advise that all quit attempts should be supported at least weekly by a trained tobacco dependence treatment advisor, which is a scarce resource in South Africa.

Nicotine replacement therapy is included in the WHO Model List of Essential Medicines as nicotine gum (2 mg and 4 mg) and transdermal patch (5 mg – 30 mg/16 h; 7 mg – 21 mg/24 h) formulations.^61^ The goal of NRT is to assist in the transition from cigarette smoking to abstinence, as replacing the nicotine obtained from tobacco alleviates nicotine withdrawal symptoms.^46^ Compared to smokers on no treatment, smokers using NRT have been reported to be almost twice as likely (with an odds ratio of 1.84) to successfully quit. Combining two or more formulations of NRT may have a synergistic effect and is possibly more effective than utilising a single NRT product.^46^

Adverse effects of NRT include nausea, hiccups, painful jaw, gastrointestinal discomfort, dizziness, sleep disturbances and a rash at the patch site. Relative contraindications for nicotine gum include dentures, peptic ulcer disease and unstable coronary artery disease.^62^

Varenicline and cytisine are classified as nicotine receptor partial agonists. They have a dual mechanism of action: the maintenance of moderate levels of dopamine to relieve nicotine withdrawal symptoms (agonist action) and reduction of reward derived from smoking (antagonist action).^63^ In a network meta-analysis of 15 comparisons, Cahill et al.^63^ found that varenicline increased smoking cessation rates by two to three times compared to placebo, with an odds ratio of 2.88.

Although safety concerns regarding varenicline use in PWMI have been expressed,^64^ there are no clearly documented pharmacokinetic interactions with psychotropics. Additionally, the evaluating adverse events in a global smoking cessation study (EAGLES) concluded that varenicline and bupropion did not significantly increase the risk of psychiatric adverse events (anxiety, depression, aggression, psychosis and suicidal behaviour) compared to placebo and NRT; in both PWMI and those without a history of mental illness.^64^

Bupropion is a noradrenaline and dopamine reuptake inhibitor, as well as a nicotinic receptor antagonist.^46^ It attempts to block or reduce the reinforcing effects of nicotine on nicotinic receptors. Bupropion use is contraindicated in seizure disorders, alcohol use disorders and eating disorders.^46^ Evaluating 36 trials in a network meta-analysis, Cahill et al.^63^ found that bupropion improved smoking cessation compared to placebo with an odds ratio of 1.82. Interestingly, five trials included participants with schizophrenia and one trial participants with PTSD.

E-cigarettes are handheld nicotine delivery devices that do not contain tobacco but rather nicotine.^46^ Nicotine dosages in e-cigarettes are markedly variable depending on the device, amount of e-liquid, components of the liquid and the frequency, size, duration and depth of inhalation.^46^ E-cigarettes appear to assist with smoking cessation more than no support or behavioural support only, based on a 2021 *Cochrane* review of 56 studies.^65^ E-cigarettes may play a role in reducing the number of cigarettes consumed and may be especially useful in chronic smokers such as patients with schizophrenia.^46,58^

However, there are concerns regarding e-cigarettes. These include the unregulated e-cigarette industry in South Africa, the tobacco industry’s ties to the e-cigarette industry, the industry’s evasion of regulations (using product diversification and advertising, etc.), undermining of tobacco control policies, a lack of evidence that they are harmless, side effects, harms of passive e-cigarette smoking, false claims that they are harmless and a lack of robust prospective clinical trials proving their efficacy.^63,66,67^

## Nonpharmacological treatments

A lack of randomised controlled trials was noted in a systematic review of psychological interventions for smoking among PWMI by Lightfoot et al. in 2020.^68^ The existing evidence indicates equal effectiveness of cognitive-behavioural therapy, motivational interviewing, behavioural counselling, personalised feedback and monitoring, compared to the general population.^68^ Key aspects relating to a positive outcome include regular, consistent sessions and a strong therapeutic alliance between the patient and practitioner.^68^

While not specific to PWMI, a community outreach intervention in India demonstrated positive effects with a 15-minutes quit advice session with a single training session in yogic breathing, compared to those receiving a one-minute quit advice session alone.^69^ In a *Cochrane* review of behavioural interventions for smoking cessation in the general population, Hartmann-Boyce et al.^70^ found that the level of support, including the number of therapeutic contacts and duration of the programme, influenced outcomes. They found good evidence for counselling and financial incentives and moderate evidence for text message and educational interventions.

The WHO Toolkit provides a guideline to primary care providers on how to practically assist tobacco users in just three to five minutes.^56^ These are summarised in the 5As below:

- **Ask**: ask all patients encountered if they use tobacco, in a nonjudgemental and empathetic way
- **Advise**: clear, strong and personalised advice to stop should be offered
- **Assess**: assess motivation to stop
- **Assist**: assist the patient in developing a STAR quit plan
  ■ Setting a quit date within two weeks
  ■ Telling others about quitting and obtaining support
  ■ Anticipating challenges
  ■ Removing tobacco products from patient’s environment
- **Arrange**: arranging a follow-up session or referral to specialist support.

The 5 Rs are aimed to increase motivation to quit in those not willing or ready to quit:

- **Relevance:** make the aim of quitting relevant to patient
- **Risks:** explain the risks of continued tobacco use
- **Rewards:** identify benefits of smoking cessation (saving money, etc.)
- **Roadblocks:** predict possible barriers or impediments to quitting
- **Repetition:** ongoing assessments of motivation should take place in subsequent consultations.

## South African guidelines

A government initiative in the South African public healthcare sector is the National Council Against Smoking.^71^ The website (https://www.againstsmoking.co.za/) includes a national quit-line, general advice regarding smoking cessation, information on NRT products and their availability as over-the-counter medications.^71^ However, while 84% of the South African population uses the public healthcare system,^72^ access to over-the-counter medications sold at private dispensaries is limited.

The 5th edition of the National Department of Health *Adult Hospital Level Standard Treatment Guidelines*^73^ recommend smoking cessation in the management of several medical conditions. However, limited guidance on how healthcare professionals should assist patients in achieving this is provided, with no psychosocial or pharmacological interventions specified.

The *South African Medical Journal* clinical practice guideline on tobacco smoking cessation^58^ emphasises a holistic approach with thorough assessment and regular follow-up and support. Pharmacological interventions (including NRT, varenicline or bupropion) are recommended for people who wish to stop smoking. Internet and telephonic resources for psychosocial interventions are provided in the guideline.

## Conclusion

Tobacco use and mental illness are highly comorbid, with possible serious public health consequences. Smoking cessation among PWMI should be considered a priority intervention. However, there is a lack of research on effective smoking cessation interventions among PWMI in South Africa. Thorough health technology assessment of biopsychosocial interventions to stop smoking among PWMI is suggested in order to determine the most affordable and sustainable methods appropriate to South Africa. In the meantime, holistic care of both mental disorders and tobacco use should be provided using available treatments at all service levels and especially in the primary care setting.
